# The Current Role of Carotid Duplex Ultrasonography in the Management of Carotid Atherosclerosis: Foundations and Advances

**DOI:** 10.1155/2012/187872

**Published:** 2012-03-07

**Authors:** Kelly R. Byrnes, Charles B. Ross

**Affiliations:** Division of Vascular Surgery and Endovascular Therapeutics, Department of Surgery, University of Louisville School of Medicine, Louisville, KY 40202, USA

## Abstract

The management of atherosclerotic carotid occlusive disease for stroke prevention has entered a time of dramatic change. Improvements in medical management have begun to challenge traditional interventional approaches to asymptomatic carotid stenosis. Simultaneously, carotid artery stenting (CAS) has emerged as an alternative to carotid endarterectomy (CE). Finally, multiple factors beyond degree of stenosis and symptom status now mitigate clinical decision making. These factors include brain perfusion, plaque morphology, and patency of intracranial collaterals (circle of Willis). With all of these changes, it seems prudent to review the role of carotid duplex ultrasonography in the management of atherosclerotic carotid occlusive disease for stroke prevention. Carotid duplex ultrasonography (CDU) for initial and serial imaging of the carotid bifurcation remains an essential component in the management of carotid bifurcation disease. However, correlative axial imaging modalities (computer tomographic angiography (CTA) and contrast-enhanced magnetic resonance angiography (CE-MRA)) increasingly aid in the assessment of individual stroke risk and are important in treatment decisions. The purpose of this paper is twofold: (1) to discuss foundations and advances in CDU and (2) to evaluate the current role of CDU, in light of other imaging modalities, in the clinical management of carotid atherosclerosis.

## 1. Introduction

Carotid atherosclerosis is one of several etiological factors for stroke, an important health problem with a high burden of disease in the western world and in developing countries. Of all strokes, an estimated 88% are ischemic in nature [1–5]. Less than 20% of these are caused by atheroma in the carotid bifurcation [6–8]. While the percentage of strokes attributed to carotid disease is relatively low, the overall social and economic burden is high. It is, therefore, important to identify and manage carotid atherosclerosis with the aim of stroke prevention.

The mortality rate for stroke in the United States has declined by nearly 70% since 1950 [9]. In December 2010, the Center for Disease Control and Prevention announced stroke was the fourth leading cause of death in the United States (down from its third place ranking which it held for decades) [10]. The identification of major risk factors through population-based studies [1, 11, 12] and randomized controlled trials (RCTs) of symptomatic [13–15] and asymptomatic [16, 17] patients has led to effective public health and clinic-based control strategies. These strategies include combining community education and targeted medical and surgical intervention in patients with increased stroke risk and have contributed, in part, to the fall in stroke mortality rates.

Diagnostic imaging has also played a central role in the clinical management of patients with carotid atherosclerosis. With the advent of vascular ultrasound in the 1980s, it became possible to identify atheroma in the carotid bifurcation noninvasively. Compared to conventional catheter-based angiography, CDU is a low-cost, low-risk, and highly portable alternative. As such, it is an attractive imaging modality for asymptomatic as well as symptomatic patients.

The technique for CDU has evolved over the years as has our understanding of the disease process. Traditionally, patients were selected for intervention based on their clinical presentation and the degree of luminal narrowing in the internal carotid artery. Modern management includes an individualized assessment of risk and takes into account brain perfusion, plaque morphology, and the presence of intracranial collateralization. These factors are essential in risk stratification and illustrate the importance of adding correlative axial imaging studies to the diagnostic work-up.

## 2. Development of CDU as a Technique

CDU is useful and accurate in the assessment of the entire spectrum of carotid atherosclerosis, from preclinical intimal-medial thickening to total internal carotid occlusion. The modality easily detects minimal disease that is not hemodynamically significant. Overestimation of mild to moderate degrees of stenoses, in fact, has been a consistent problem [18]. Nevertheless, any test intended for screening must have a high degree of sensitivity to be used in the initial assessment of disease.

Hemodynamically significant stenosis is diagnosed primarily through the measurement of markedly elevated flow velocities using spectral Doppler, one component of CDU, in the narrowed portion of the internal carotid lumen. The increase in velocity is proportional to the severity of the obstruction. To diagnose stenosis, measurements of peak systolic velocity (PSV) are compared to velocity thresholds derived from correlations with conventional angiography. Secondary parameters for quantifying stenosis include end-diastolic velocity, internal carotid artery (ICA) to common carotid artery (CCA) ratio, degree of spectral broadening, and presence of plaque on B-mode imaging. Other factors to consider when interpreting studies include the presence of calcific plaque (with acoustical shadowing that may limit visualization), contralateral high-grade stenosis, and kinking or bends in the vessel (which may falsely elevate velocities).

The initial work into establishing clinically relevant thresholds was done by investigators at the University of Washington. They developed broad categories of stenosis (Table 1) as follows: 1–15%, 16–49%, 50–79%, 80–99%, and occluded [19]. These original criteria laid the foundation for CDU interpretation of carotid artery stenosis. In 2003, the criteria were modified following the results of several clinical trials involving patients typically referred for CDU [20].

The Asymptomatic Carotid Atherosclerosis Study (ACAS) was a landmark prospective, randomized clinical trial which examined the role of carotid endarterectomy in patients without symptoms (but with clinical markers for atherosclerosis). ACAS randomized 1662 asymptomatic patients with a 60% or greater ICA stenosis to either medical therapy alone or to medical therapy plus CE. In 1995, this study reported CE drastically reduced the estimated risk of ipsilateral stroke or death from 11% to 5.1% [16]. Symptomatic patients (presenting with a neurologic event such as a transient ischemic attack (TIA), stroke, or amaurosis fugax) formed the basis for such well-known studies as the North American Symptomatic Carotid Endarterectomy Trial (NASCET) [13] and the European Carotid Surgery Trial (ECST) [14]. Both trials reported a clear surgical benefit in patients with ≥70% stenosis. As a result of the RCTs, it was determined that the utility of CDU could be increased by redefining thresholds to identify patients (asymptomatic or symptomatic) with ≥60–99% stenosis. Thus, the criteria for carotid artery stenosis were modified (Table 2) by a panel of experts to be compatible with what were then accepted indications for CE, based on trial results [20]. 

AbuRahma et al. [24] used the criteria proposed by the consensus panel to analyze the correlation between CDU and angiography in 376 internal carotid arteries at their institution. They demonstrated a sensitivity of 93%, specificity of 68%, and overall accuracy of 85% for carotid stenosis between 50 and 69%. Using a cutoff PSV of ≥230 cm/s for ≥70% stenosis, they demonstrated a sensitivity of 99%, specificity of 86%, and overall accuracy of 95%. Individual vascular laboratories may have varying degrees of sensitivity and specificity based on different threshold criteria that have been internally validated. Internal validation of Doppler thresholds is recommended, but this may be difficult given the infrequency of correlative angiograms at most institutions [20]. In the absence of internal validation, the consensus panel criteria should be used for CDU interpretation.

The technique of CDU continues to evolve as a result of experience and advances in technology. Experience has been gained through research, continuing education, and establishment of quality standards through vascular lab accreditation and credentialing of sonographers and interpreting physicians. Advances in technology include continued improvements in gray-scale resolution, Power Doppler, and computer-assisted normalization of images to aid in the evaluation of plaque surface and structure characteristics [18]. No longer just a diagnostic tool, CDU guides intervention in modern practice and plays an integral role in the management of patients with carotid atherosclerosis.

## 3. Clinical Management of Carotid Atherosclerosis

For nearly half a century, the management of carotid atherosclerosis has been dictated by the severity of disease (percent stenosis) and classification of clinical presentation between “symptomatic” and “asymptomatic”. The design and results of previous clinical trials on surgical versus medical treatment of carotid atherosclerosis for stroke prevention as well as current studies comparing CE and CAS have led to this distinction. Currently, an individual's risk of stroke can be assessed by taking into account supplementary diagnostic information such as plaque morphology and the integrity of intracranial collateralization. This information identifies plaque vulnerable to disruption and atheroembolization leading to a better calculation of an individual's risk for stroke. The recognition of vulnerable plaque is also paramount in the surgical management of carotid atherosclerosis, particularly when CAS is planned. 

Carotid plaques with a large lipid core and thin fibrous cap are more likely to rupture [31]. When the area of plaque occupied by lipid components (macrophages and extracellular lipids) is >40% of the area occupied by fibromuscular components (smooth muscle cells and collagen), the plaque is considered unstable [32]. A thin fibrous cap is the result of increased collagen degradation and decreased collagen formation and is more susceptible to disruptive hemodynamic forces [33]. 

Large pressure gradients across a stenosis can influence plaque by increasing the wall shear stress (WSS) and causing cap disruption [34, 35]. Li et al. have reported that WSS rises with increasing severity of stenosis [34]. The degree of luminal narrowing, however, is just one factor contributing to large pressure drops. Lal [36] recently studied the effect of incomplete intracranial collateralization on carotid flow rates and velocities and found the pressure drop across a similar stenosis was significantly higher for an incomplete circle of Willis (CoW) compared with an intact CoW. In their model, a carotid stenosis of 67%, when associated with an incomplete CoW, contributed to markedly elevated WSS, well beyond the threshold predictive of plaque rupture.

The identification of additional parameters contributing to an individual's risk of stroke may warrant modifications to the diagnostic work-up for carotid atherosclerosis. The current role of CDU and correlative axial imaging (CE-MRA and CTA) is outlined in Table 3 and discussed further in the next section.

### 3.1. Current Role of CDU

The sensitivity and accuracy of CDU in the detection of hemodynamically significant stenosis has led to its widespread use in the initial evaluation of patients with neck bruits (and clinical risk factors for atherosclerosis) and in symptomatic patients for the detection of ≥70% carotid artery stenosis. But, the relative low specificity of CDU, especially in the 50–69% category (Table 3), justifies the use of additional imaging for positive selection of patients prior to undertaking any interventions. This is of particular relevance in the clinical management of asymptomatic patients with ≥60% stenosis, since as many as 89% of these individuals remain stroke-free with medical therapy alone [16].

While conventional angiography is still considered the “gold standard” for defining carotid disease, there has been a growing interest in performing CE based on clinical evaluation and CDU alone. One reason for this strategy is to improve patient outcomes. There is a higher risk of stroke (10–20%) within the first 14 days following a cerebrovascular event. After this time, the stroke risk declines to that of the “asymptomatic” stenosis (1-2%/year) [37]. To maximize the benefit of either CE or CAS, rapid assessment and early intervention are needed. CDU is readily accessible and allows the fastest time to identify patients in need of invasive therapy.

This trend has also been stimulated by improvements in the accuracy and utility of CDU, along with increasing demands to minimize both the risks and cost of medical care. In many centers, carotid angiography is no longer done routinely, even when planning intervention, to eliminate the procedural risk of neurologic events, which is around 1% [38, 39]. A survey of panelists convened for the Consensus Criteria conference found as many as 80% of patients in the United States undergo CE after a CDU as the only preoperative imaging study [20]. Given the specificity of 68–86% [24] in CDU detection of stenosis above the 60% threshold, the practice of undertaking CE without additional imaging suggests that unnecessary procedures will be performed. When CE appears indicated by CDU, confirmatory imaging with CE-MRA should be considered. If there is discordance between the two imaging modalities, then CTA would be a suitable arbiter. It should be noted that multiple imaging modalities may unfavorably delay treatment when imaging resources are limited.

**Figure 1:**
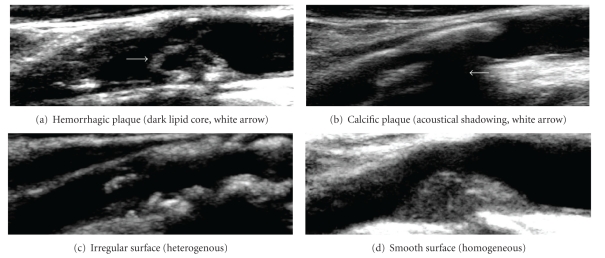
CDU and plaque morphology (structure and surface characteristics).

With the emergence of CAS as an alternative to CE, there has been an increased focus on CDU's ability to distinguish plaque morphology. Plaque morphology includes surface (smooth versus irregular) and structure (homogeneous, heterogeneous, hemorrhagic, calcified, echolucent) characteristics (Figure 1). Certain plaque characteristics can affect patient outcomes. For example, echolucency within a carotid plaque correlates with the presence of lipid components and is associated with increased neurologic events [38–40] as are plaques that are heterogeneous and/or irregular as opposed to smooth and homogeneous [41]. This is a notable concern in CAS since plaque is not removed but rather pushed to the side. The Imaging in Carotid Angioplasty and Risk of Stroke (ICAROS) study confirmed the relationship between echolucent plaques and the risk of stroke during CAS, as well as CDU's ability to distinguish such plaques, through a multicenter registry [42].

Individual risk assessment of patients being considered for surgical intervention is limited with CDU. For example, the assessment of plaque morphology is not routinely available in every vascular lab and requires specific protocols to assure standardization of results. The use of image normalization and software calculation of gray-scale median values can minimize inter- and intraobserver variability, but is not the current standard of practice. CDU also fails to provide diagnostic information with regard to brain perfusion, arch pathology, intracranial collateralization, and vascular anomalies such as aneurysms. The CoW can be interrogated with transcranial Doppler, although few labs employ this technique.

### 3.2. Surveillance with CDU following CAS and CE

Recurrent stenosis is one of the most prevalent complications of CAS. The current risk of hemodynamically significant (≥80%) in-stent restenosis (ISR) is 6.4% at 5 years [38]. Because of the possibility of ISR and the importance of late stroke prevention following CAS, strict follow-up and surveillance with CDU are necessary. The same technique used in preoperative assessment is applied. However, it is important to note that previously published velocity criteria for CDU were based on native, nonstented arteries. Blood flow and vessel compliance are altered in stented arteries, and the use of existing criteria may overestimate the degree of restenosis [28, 39–43]. Modified velocity criteria thresholds proposed by Lal et al. [29] and AbuRahma et al. [28] (Table 4) correlate with clinically significant ISR following CAS and should be used for CDU surveillance in these patients. 

ISR is primarily caused by neointimal hyperplasia. The course of neointimal hyperplasia is difficult to predict; it may progress to a high-grade stenosis requiring reintervention or follow a more benign course. Lal et al. [44] have studied patterns of ISR and developed a classification scheme based on the length of the lesion and its relationship to the stent which is predictive of the need for future remedial intervention. Lesions >10 mm long with extension beyond the stent(s) margins were most worrisome and required remedial intervention by transcatheter techniques in 58.8% of their cases. 

A full discussion of ISR is beyond the scope of this paper. What is important is that CDU is a reproducible technique that can be applied to the identification and classification of types and severity of ISR. CDU surveillance should include a thorough investigation of the stented segment noting the length of any narrowing and its relationship to the stent. Standard practice is to obtain baseline CDU prior to discharge following CAS. Follow-up CDUs are obtained at 3 months and then at 6-month intervals for the next 18 months. If there is no evidence of significant ISR at two years, surveillance CDUs are then performed annually [45]. Since long-term outcomes are still not well defined, lifelong surveillance with CDU is recommended. 

CDU surveillance following CE, on the other hand, is a topic of controversy. Stroke prevention is the goal of any surveillance program. Strokes can be prevented by detection of significant restenosis prior to the onset of neurologic events and through follow-up of contralateral carotid bifurcation disease. Opponents to routine surveillance argue that there is no real stroke prevention benefit. While recurrent stenosis is somewhat common after CE with a reported incidence of 1% to 37%, fewer than 8% of patients become symptomatic [46–48]. Restenosis rates are also affected by the type of closure (primary versus patch angioplasty) with lower rates favored in the patch angioplasty group [49]. The clinical significance of carotid restenosis following CE has led some investigators to conclude that postoperative CDU is not warranted. Current recommendations for CDU surveillance following CE with primary closure are 1 month, 6 months, and yearly [8]. For CE with patch closure, if CDU is normal at 6 months (and there is no contralateral disease), routine follow-up may not be necessary [50].

### 3.3. Routine CDU Screening for Asymptomatic Carotid Atherosclerosis

Routine screening of the general asymptomatic population is controversial. The United States Preventive Services Task Force (USPSTF) recommended against routine screening for asymptomatic carotid artery stenosis in 2007. It concluded it was not possible to identify people from a high-risk group (with a prevalence of 5%) who might benefit from screening and treatment with CE or CAS [51]. 

Similarly, new guidelines on the management of patients with carotid artery disease were released in January 2011 by the American College of Cardiology Foundation/American Heart Association Task Force on Practice Guidelines. Specific to screening, the task force recommended against screening in asymptomatic patients without significant risk factors for atherosclerosis or physical signs of carotid disease. However, screening for carotid stenosis “may be considered among individuals with at least two major risk factors for atherosclerosis or with a diagnosis of other cardiovascular disease such as coronary artery disease or peripheral artery disease” [8]. 

Screening in high-risk groups has also been reported in Hong Kong. Cheng et al. [52] used CDU to screen for asymptomatic carotid artery stenosis in a group of elderly (mean age 70.6 years) Chinese patients with known lower extremity arterial disease. The prevalence of severe (≥70%) internal carotid artery stenosis in this group was 24.7%. Age, smoking quantity, and a carotid bruit were independent risk factors associated with severe carotid disease. The degree of carotid stenosis also correlated with age and the number and duration of cigarette smoking. This study suggests it may be possible to identify a group of patients with a high prevalence of carotid stenosis that might benefit from screening and treatment with CE or CAS. 

## 4. Emerging Advances in CDU

Atherosclerosis is a systemic disease with potentially devastating consequences such as stroke, ischemic heart disease, and peripheral arterial disease. Emerging advances in carotid ultrasound allow for the detection of atherosclerosis at its earliest stages. These advances include the use of carotid intima-media thickness (CIMT) calculations and administration of contrast ultrasound agents during CDU. 

**Figure 2:**
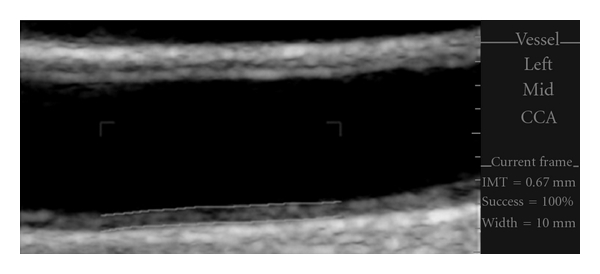
Carotid IMT. Automated edge detection software used to measure CIMT in the mid-common carotid artery (CCA). The CIMT was calculated at 0.67 mm in this patient.

The application of CIMT has become an accepted, reliable surrogate marker for determination of atherosclerosis and is endorsed by the U.S. Food and Drug Administration (FDA) and the European Agency for the Evaluation of Medicinal Products [53, 54]. CIMT measurements correlate well with histology, and increased IMT (Figure 2) is associated with cardiovascular risk factors and the presence of more advanced atherosclerosis. CIMT has led to improved cardiovascular risk stratification. Nambi et al. [53] used the Atherosclerosis Risk in Communities (ARIC) database to correlate cardiovascular events to CIMT. In the intermediate-risk group, 21.7% of participants were correctly reclassified following the addition of CIMT to traditional risk factors. This is an improvement to the widely accepted Framingham Risk Score. 

Contrast-enhanced ultrasound (CEUS) is another emerging advancement in technology. While not routinely used, contrast agents are an exciting adjunct to CDU and have two potential benefits. First, they can be used to identify neovascularization within the adventitial layer of the CCA which precedes the development of increased IMT [25, 55]. Clinical application includes management of atherosclerosis at its earliest stage of development. Second, CEUS can be used to quantify plaque morphology by identifying intraplaque neovascularization [56]. There is a direct correlation between intraplaque neovascularization and cardiovascular events (myocardial infarction, TIA, stroke) [25, 55–57]. Consequently, CEUS has an emerging role in the selection of patients for carotid intervention. 

Despite the abundance of the literature on the benefit of advanced noninvasive technologies in the assessment of cardiovascular risk, their role is still emerging. In the United States, CIMT and CEUS are not generally reimbursable. Additionally, contrast ultrasound agents are currently FDA approved only for use in the imaging of cardiac structures. Their off-label use in vascular imaging may require institutional approval. In developing countries, emerging advances in CDU may be of greater immediate benefit. This is especially true of CIMT which can be measured relatively simply and is well-suited for large-scale population studies.

## 5. Conclusion

The role of CDU in the management of carotid atherosclerosis has evolved. The technique was developed as a diagnostic tool to identify asymptomatic and symptomatic patients with significant (≥60–99%) carotid stenosis. These were the patients likely to benefit from carotid revascularization either by CE or CAS. Today, correlative axial imaging (CE-MRA and CTA) studies supplement CDU and provide diagnostic information on brain perfusion, plaque morphology, and intracranial collateralization. These factors are important in the individual assessment of stroke risk and may improve the clinical management of patients with carotid atherosclerosis.

## Figures and Tables

**Table 1:** University of Washington criteria [19].

University of Washington (Strandness)			
Stenosis (%)^a^	PSV^b^ (cm/s)	EDV^c^ (cm/s)	Flow characteristics
1–15	<125	<140	No spectral broadening
16–49	<125	<140	Minimal spectral broadening
50–79	≥125	<140	Marked spectral broadening
80–99	≥125	>140	Marked spectral broadening
Occlusion	N/A	N/A	No internal carotid flow signal

^a^Based on conventional angiography using least transverse diameter at the stenosis compared to the diameter of the distal uninvolved ICA where the arterial walls become parallel, ^b^peak systolic velocity, ^c^end diastolic velocity.

**Table 2:** Carotid Consensus Panel criteria [20].

Carotid Consensus Panel criteria (2003)			
Stenosis (%)^a^	PSV^b^ (cm/s)	EDV^c^ (cm/s)	ICA/CCA ratio
Normal (no plaque)	<125	<40	<2.0
<50 (plaque seen)	<125	<40	<2.0
50–69	125–230	40–100	2.0–4.0
≥70	≥230	>100	>4.0

^a^Based on conventional angiography using least transverse diameter at the stenosis compared to the diameter of the distal uninvolved ICA where the arterial walls become parallel, ^b^peak systolic velocity, ^c^end diastolic velocity.

**Table 3:** Comparison of diagnostic imaging modalities for clinical management of carotid atherosclerosis.

							Diagnostic imaging modality								
			CDU				CE-MRA				CTA				
			Tends to overestimate moderate (50–69%) stenosis (i) Good for screening tool (ii) Prior to surgical intervention, obtain CE-MRA imaging for positive selection, especially in asymptomatic patients [21, 22]				Tends to overestimate moderate (50–69%) stenosis (i) Specificity in this category is improved when there is concordance with CDU				Highest specificity and overall accuracy of the three imaging modalities [22, 23] Combining data sets with submillimeter spatial resolution with dedicated MPR reconstructions obtained in oblique planes or parallel to the vessel lumen provides a better evaluation of percent stenosis				
	% Stenosis		Consensus criteria (AbuRahma et al.) [24]:				Anzidei et al. [22]:				Anzidei et al. [22]:				
			% Stenosis	Sensitivity	Specificity	Accuracy	% Stenosis	Sensitivity	Specificity	Accuracy	% Stenosis	Sensitivity	Specificity	Accuracy	
			50–69%	93%	68%	85%	≥70%	93%	97%	95%	≥70%	95%	98%	97%	
			≥70%	99%	86%	95%									
Selection of patients for intervention		Plaque morphology	Not routine in every vascular lab and requires specific protocols to assure standardization of results: (i) B-mode (surface and structure characteristics) (ii) Contrast [25] (a) Neovascularization of adventitia (earliest stage of atherosclerosis) [25] (b) Intraplaque neovascularization (vulnerable plaque) (ii) Grayscale median (GSM) calculation (using Adobe Photoshop) [26] (a) ≤25 increased risk for embolization				When dedicated protocols are used, CE-MRA can demonstrate specific plaque components, including calcium, lipid, fibrocellular element, or thrombus. It can also distinguish between an intact (thick, thin) or ruptured fibrous cap [22]				Able to discriminate between lipid components, fibrous components, and the calcium present in atheromas [22]				
	Individual risk assessment	Intracranial	(i) Integrity of CoW can be assessed with TCD/TCI (not routinely done in all vascular labs) (ii) Unable to assess other intracranial pathology				Well suited for delineating intracranial anatomy (CoW, aneurysms, tandem lesions)				Well suited for delineating intracranial anatomy (CoW, aneurysms, tandem lesions)				
		Brain perfusion	Time-intensity curves with TCD and contrast agent (not routine) [27]				With addition of MRI				Can be done at same time as CTA				
Surveillance following intervention		CE	Low cost and low risk of this imaging modality make it ideal for postprocedure follow-up. Primary closure: (i) <1 month, 6 months, yearly Patch closure: (i) 6 months (if normal, and there is no contralateral disease, routine follow-up may not be necessary) (ii) Modified CDU criteria may be necessary				May have role if restenosis detected by CDU or patient is symptomatic				May have role if restenosis detected by CDU or patient is symptomatic				
		CAS	Low cost and low risk of this imaging modality make it ideal for postprocedure follow-up. Modified CDU criteria are necessary (blood flow and vessel compliance are altered in stented arteries) [28, 29] Strict follow-up and surveillance for in-stent restenosis (ISR): (i) Baseline (before discharge), 3 months, and every 6 months for first 18 months (ii) If no significant ISR at 2 years, then perform annually (with lifelong surveillance since long-term outcomes are not well defined)				Varying stent designs and related artifacts limit widespread use [30]				Need for frequent follow-up makes use of ionizing radiation and nephrotoxic contrast unsuitable				

**Table 4:** Comparison of modified criteria for ISR after CAS.

Modified criteria for ISR after CAS		
Stenosis (%)^a^	PSV^b^ (cm/s)	ICA/CCA ratio
Lal et al. (2008) [29]		

**0–19 **	<150	<2.15
**20–49 **	150–219	
**50–79 **	220–339	≥2.7
**80–99 **	>340	≥4.15

AbuRahma et al. (2008) [28]		

**0–29**	<154	<1.5
**30–49**	154–223	
**50–79**	224–324	≥3.4
**80–99**	>325	≥4.5

^a^Based on conventional angiography using least transverse diameter at the stenosis compared to the diameter of the distal uninvolved ICA where the arterial walls become parallel, ^b^peak systolic velocity.
